# Case Report: About a Case of Hyperammonemia Syndrome Following Lung Transplantation: Could Metagenomic Next-Generation Sequencing Improve the Clinical Management?

**DOI:** 10.3389/fmed.2021.684040

**Published:** 2021-07-06

**Authors:** Charlotte Michel, Michela Raimo, Vladimir Lazarevic, Nadia Gaïa, Nina Leduc, Christiane Knoop, Marie Hallin, Olivier Vandenberg, Jacques Schrenzel, David Grimaldi, Maya Hites

**Affiliations:** ^1^Department of Microbiology, Laboratoire Hospitalier Universitaire de Bruxelles, Université Libre de Bruxelles, Brussels, Belgium; ^2^Clinic of Infectious Diseases, Cliniques Universitaires de Bruxelles, Erasme Hospital, Brussels, Belgium; ^3^Genomic Research Laboratory, Geneva University Hospitals, Geneva, Switzerland; ^4^Department of Pneumology, Cliniques Universitaires de Bruxelles, Erasme Hospital, Brussels, Belgium; ^5^Intensive Care Unit, Cliniques Universitaires de Bruxelles, Erasme Hospital, Brussels, Belgium

**Keywords:** metagenomics, mollicutes, hyperammonemia, lung trasnplantation, next-generation sequencing

## Abstract

**Background:** *Mycoplasma hominis* and *Ureaplasma* spp. are responsible for opportunistic infections in transplant patients, sometimes causing a life-threatening hyperammonemia syndrome. Both pathogens are not identified with standard microbiology techniques, resulting in missed or delayed diagnosis. We present a clinical case that illustrates the added value that next-generation sequencing (NGS) may offer in the diagnosis of respiratory infections in immune-compromised patients.

**Results:** A 55 years-old man with idiopathic pulmonary fibrosis underwent double lung transplantation. He received antibiotic prophylaxis with piperacillin-tazobactam and azythromycin. At day 4 post-transplantation (PTx), the patient presented an acute respiratory distress. A broncho-alveolar lavage (BAL) was performed. At day 5 PTx, the patient presented a status epilepticus due to diffuse cerebral oedema. Serum ammonia concentration was 661 μg/dL. BAL bacterial culture was negative. Because of the clinical presentation, special cultures were performed and identified 100.000 CFU/mL of *M. hominis* and *Ureaplasma* spp. and specific PCRs were positive for *M. hominis* and *Ureaplasma parvum*. Antibiotic therapy was shifted to therapeutic dose of azithromycin and doxycycline; within 48 h ammonia serum concentrations returned to normal but the coma persisted several weeks, followed by a persistent frontal lobe syndrome. A follow-up BAL was performed on day 11 Ptx. The *Mycoplasma/Ureaplasma* culture was negative, yet the specific PCRs remained positive. Bacterial culture found 100 CFU/mL of *Staphylococcus aureus* and viral culture was positive for Herpes Simplex Virus-1. These results were confirmed by metagenomic next-generation sequencing (mNGS). In the bacterial fraction, the majority of reads belonged to *Corynebacterium propinquum* (34.7%), *S. aureus* (24.1%) and *Staphylococcus epidermidis* (17.1%). Reads assigned to *M. hominis, Ureaplasma urealyticum* and *parvum* represented 0.71, 0.13, and 0.04% of the bacterial fraction and corresponded to 6.9 × 10^3^, 9.7 × 10^2^, and 3.7 × 10^2^ genome equivalents per mL of BAL fluid, respectively. These results are in favor of a cure of the atypical infection.

**Conclusions:** mNGS offered added diagnostic and quantitative values compared to PCR tests, which can remain positive after resolved infections. The initiation of appropriate antibiotic therapy would have occurred earlier on, possibly resulting in a better clinical outcome if mNGS had been performed in a routine fashion.

## Background

We report here a clinical case that illustrates the limitations of both culture-dependent and molecular routine microbiological methods and the potential added value of metagenomic next-generation sequencing (mNGS) in the diagnosis of respiratory infections in immunocompromised patients.

## Case Report

A 55-year-old man with idiopathic pulmonary fibrosis, and treated since 2016 with pirfenidone, underwent a double lung transplantation in 2019. The patient had no significant medical history other than dyslipidemia treated with statins. During surgery, the patient presented an episode of ventricular fibrillation with cardiac arrest due to the passage of air bubbles when the right pulmonary artery was unclamped. He was quickly resuscitated after cardioversion.

The patient received a piperacillin-tazobactam and azithromycine antibiotic prophylaxis and a classic immunosuppressive regimen (Thymoglobulin, mycophenolic acid, and methylprednisolone). On day 4 post-transplantation, he presented an acute respiratory distress that required reintubation. As the perioperative swab culture from the donor lungs revealed *Escherichia coli* of intermediate susceptibility to piperacillin-tazobactam, an *E. coli* pneumonia was suspected, and piperacillin-tazobactam was shifted to cefuroxime. A broncho-alveolar lavage (BAL) was performed before antibiotic shift and sent for bacterial cultures, galactomannan measurement, and detection of 34 pulmonary pathogens by multiplex polymerase chain reaction (PCR) (TaqMan Array Card, Thermo Fisher Scientific, Waltham, MA, USA), which only revealed the presence of Herpes simplex virus type 1 (HSV-1) with a coefficient threshold at 34, suggesting no acute infection. At day 5 post-transplantation, fasciculations of the facial muscles appeared. The patient's parameters did not reveal malignant hypertension. A continuous electroencephalogram (EEG) revealed a status epilepticus, and the patient was treated by levitiracetam and midazolam. Computed tomography revealed the presence of a diffuse brain edema, therefore excluding the possibility of performing a spinal tap. Blood tests revealed severe hyperammonemia at 661 μg/dL; an aggressive treatment was initiated with lactulose, L-carnitine, sodium benzoate, sodium-phenylbutyrate, and continuous veno-venous hemodiafiltration (CVVH), as well as restriction of protein intake. A treatment with doxycycline was also initiated, and the dosage regimen of azithromycin was increased from 250 mg, three times weekly to 500 mg daily, the following day.

The “standard” bacterial culture of the BAL fluid revealed negative results. Since clinical features suggested an infection by atypical pathogens, a specific culture for *Mycoplasma*/*Ureaplasma* spp. was performed, identifying 100,000 CFU/mL of *Mycoplasma hominis* and *Ureaplasma* spp. after 24 h of incubation. Because of the small size of colonies, it was impossible to obtain pure cultures, and their identification relied on microscopic examination. Antibiotic susceptibility testing of a mixed population was challenging, and an accurate minimal inhibitory concentration (MIC) could not be determined with the Mycoplasma IST2 susceptibility gallery (bioMérieux, Marcy l'Etoile, France). The mixed culture was interpreted as resistant to erythromycin, clindamycin, and azithromycin but susceptible to tetracycline, doxycycline, ofloxacin, and ciprofloxacin. Ammonia serum concentrations returned to normal values (<102 μg/dL) within 48 h of initiating doxycycline treatment. The coma persisted several weeks and was followed by a severe, irreversible frontal lobe syndrome and massive cognitive impairment. The patient is at present (24 months after transplantation) bedridden.

To confirm microbiological cure, a follow-up BAL was performed on day 12 post-transplantation, after 7 days of doxycycline and 5 days of azithromycin. Bacterial culture yielded 100 CFU/mL of *Staphylococcus aureus*, and both multiplex PCR for respiratory pathogens (Taqman Array Card) and viral culture were positive for HSV-1, all of which were not considered clinically significant. The specific culture for *Mycoplasma*/*Ureaplasma* spp. revealed negative results. The antibiotic therapy was stopped after a total of 10 days of doxycycline and 8 days of azithromycin at therapeutic doses.

**Figure 1:**
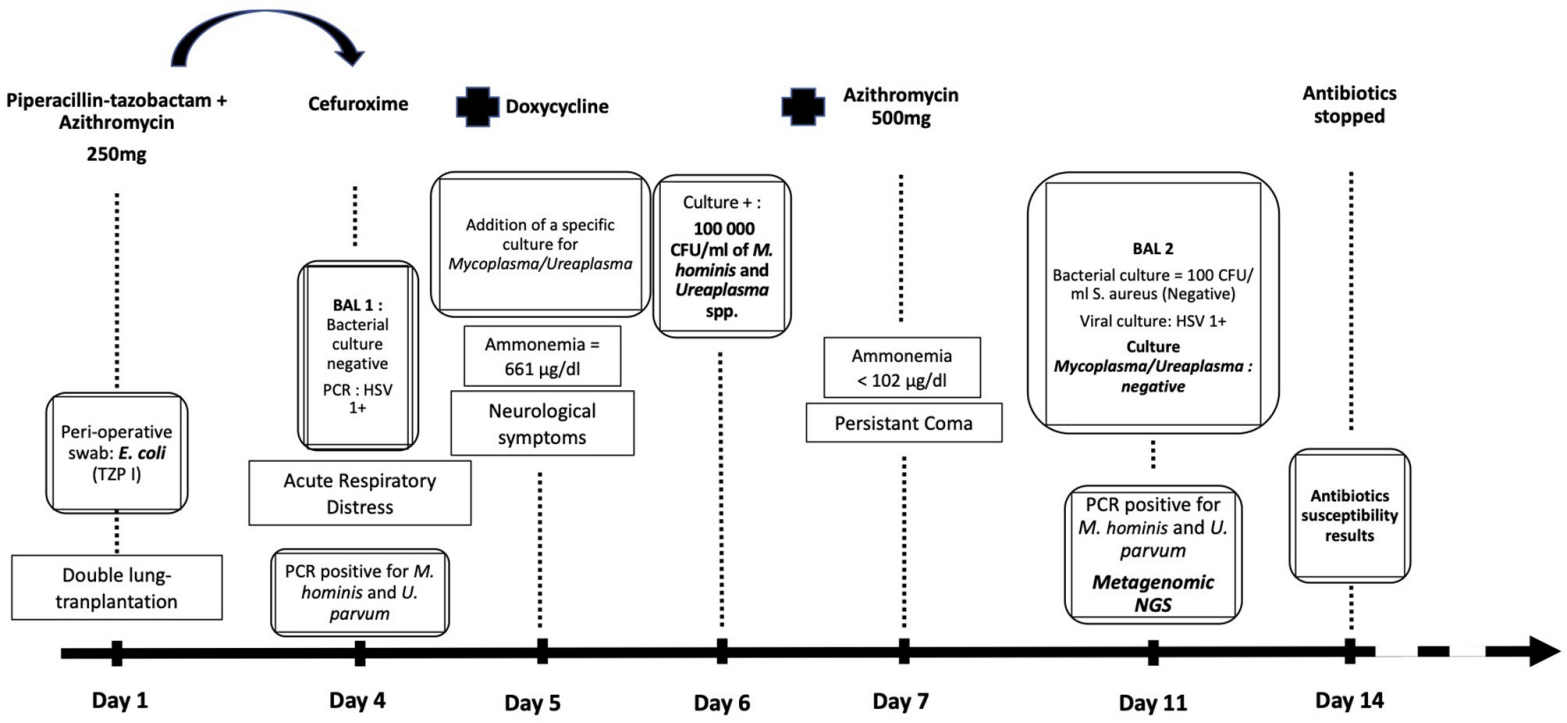
Chronology of diagnostic and treatment of the patient according to the laboratory results. Apparition of the symptoms through time, Day 1 corresponding to the lung-transplantation. Antibiotic therapy shifts. Ammonemia levels. Results of microbiological cultures and biomolecular tests.

A retrospective analysis was performed by specific qualitative PCR for species identification and revealed *M. hominis* and *Ureaplasma parvum* in both BAL samples. mNGS was performed for scientific documentation on the second BAL sample (unfortunately, no material was left from the first BAL sample). Of 2,275,532 quality filtered sequencing reads, 2,264,070 were human, 4,222 mapped to HSV-1, and 4,773 were assigned by CLARK to bacteria. In the bacterial fraction, the majority of reads belonged to *Corynebacterium propinquum* (34.7%), *S. aureus* (24.1%), and *Staphylococcus epidermidis* (17.1%). Reads assigned to *M. hominis, Ureaplasma urealyticum*, and *U. parvum* represented 0.71, 0.13, and 0.04% of the bacterial fraction and corresponded to 6.9 × 10^3^, 9.7 × 10^2^, and 3.7 × 10^2^ genome equivalents per mL of BAL fluid, respectively (Table 1). BLASTn analysis confirmed CLARK assignments, with the exception of one read pair that was classified by CLARK as *U*. *parvum* but had best BLASTn scores to *M*. *hominis*. These results are in favor of a cure of the atypical infection. The chronology of the case is represented in Figure 1.

**Table 1:** Microbial species detected by mNGS.

**Species identified by CLARK**	**Read (pair)** **count**	**Genome equivalents** **per mL of** **BAL fluid**	**Genome** **coverage (%)**
Human alphaherpesvirus 1 (HSV-1)	4,222	3.9E+06	91.46
*C. propinquum*	1,658	9.3E+04	11.9
*S. aureus*	1,151	5.7E+04	9.51
*S. epidermidis*	816	4.6E+04	7.21
*Corallococcus aberystwythensis*	180	2.5E+03	0.04
*Streptomyces turgidiscabies*	141	1.9E+03	0.01
*Corynebacterium pseudodiphtheriticum*	87	5.4E+03	0.89
*Bifidobacterium tibiigranuli*	77	4.0E+03	0.03
*Staphylococcus schweitzeri*	68	3.4E+03	0.79
*Staphylococcus argenteus*	50	2.6E+03	0.51
*Mogibacterium diversum*	45	3.5E+03	0.54
*Enterococcus faecalis*	42	2.1E+03	0.33
*[Candida] glabrata*	40	4.6E+02	0.07
*Alcanivorax hongdengensis*	35	1.3E+03	0.02
*M. hominis*	34	6.9E+03	1.08
*Aequorivita lutea*	27	1.1E+03	0.05
*Sphingomonas paucimobilis*	18	6.3E+02	0.09
*Parvimonas micra*	13	1.1E+03	0.17
*Porphyromonas endodontalis*	12	8.0E+02	0.12
*Staphylococcus haemolyticus*	11	6.1E+02	0.1
*Lancefieldella parvula*	10	9.1E+02	0.13
*Peptostreptococcus stomatis*	8	5.7E+02	0.07
*Bifidobacterium dentium*	8	4.3E+02	0.05
*Staphylococcus warneri*	7	4.1E+02	0.07
*Cutibacterium acnes*	7	3.9E+02	0.06
*U. urealyticum*	6	9.7E+02	0.15
*Gemella morbillorum*	5	4.0E+02	0.06
*Mogibacterium timidum*	5	3.8E+02	0.06
*Tropheryma whipplei*	3	4.6E+02	0.07
*U. parvum*	2	3.7E+02	0.05

*Only microorganisms with >3E+02 genome equivalents per mL of BAL fluid are reported*.

## Discussion

*Mycoplasma* and *Ureaplasma* (class Mollicutes) have no cell wall and therefore are undetectable under light microscopy after Gram staining and are intrinsically resistant to antibiotics targeting peptidoglycans. They also cannot be grown on standard bacterial culture media. *M. hominis* and *Ureaplasma* spp. are urogenital commensals, which are long known to be responsible for infections of the urogenital tract. They also have been found in a wide panel of opportunistic infections, such as periaortic abscess, pneumonia, and pericarditis (1–3). Since 2015, *M. hominis, U. urealyticum*, and *U. parvum*, alone or in co-infection, have been increasingly documented as causes of hyperammonemia syndrome (HS) within the first 30 days after lung or hematopoietic cell transplantation (3–7). HS occurs rarely (1–4% of transplant patients) but has major impact in terms of neurological complications (3, 4) and mortality. The mortality rate in patients with increased ammonia levels is 67% compared to 17% in patients with normal ammonia levels (4).

*Ureaplasma* spp. have the ability to generate energy by cytosolic urease-catalyzed hydrolysis of urea to ammonia. This has no clinical consequences in urogenital infections, but it can be severe in disseminated infections: after the serum ammonia level increases, the liver converts it back to urea, which in turn provides more energy for the microorganism, resulting in sustained hyperammonemia (3, 8). To investigate the source of opportunistic infections caused by *Ureaplasma* in lung-transplant patients, Fernandez et al. screened all lung donors. The study found a significant correlation between *Ureaplasma*-positive lung donors and hyperammonemia (*p* < 0.001). Additionally, all recipients of *Ureaplasma*-positive lungs presented a systemic inflammatory response, with lung infiltrates and required vasopressors to maintain a normal blood pressure after transplantation (7).

*Mycoplasma/Ureaplasma* spp. are naturally susceptible to macrolides, tetracyclines, or fluoroquinolones (9), but these antibiotics are not part of the classic antibiotic prophylaxis regimen used for lung transplantation (10). Acquired resistance in these bacteria is, however, not unusual, even though resistance rates vary significantly between studies. A French study showed resistance rates to tetracyclines of 7.5% for *Ureaplasma* spp. and 14.8% for *M. hominis* isolates, while the levofloxacin and moxifloxacin resistance rates were, respectively, 1.2 and 0.1%, for *Ureaplasma* spp., and 2.7 and 1.6% for *M. hominis* (11). An Italian study, on the other hand, showed significant levels of quinolone resistance in *Ureaplasma* spp., particularly for ciprofloxacin (77% of cases). Furthermore, *M. hominis* strains were non-susceptible to azithromycin and roxithromycin in about 90% of cases (12).

The increasing number of immuno-suppressed patients is one of the reasons for the emergence of infections caused by new opportunistic, fastidious or yet-to-be-cultured pathogens. The development of culture-independent diagnostic microbiology tools is essential to improve the healthcare of these patients. Our patient, for instance, suffered a delayed microbiological diagnosis and late documentation of the susceptibility profile, resulting in irreversible neurological complications, despite appropriate empirical antibiotic therapy initiated at the presentation of symptoms.

The specific qualitative PCRs for *M. hominis* and *U. parvum* were positive on the first and the second BAL. The first one allowed an accurate diagnosis to the species level but the second remained positive even after the microbiological cure of the patient. The positivity of a PCR can last after the patient is “cured” due to the presence of the pathogen's cell-free DNA, dead cells, and viable but not culturable cells. The procedure we used to extract DNA for mNGS included specific pretreatments aimed at removing free DNA and DNA from human and dead or damaged bacterial cells. Thus, the detected bacteria are expected to mostly correspond to viable populations, which, however, may contain non-culturable cells, as reported for *M*. *hominis* (13).

In our case, the results of mNGS performed on the follow-up BAL were in accordance with those found by bacterial and viral identifications by culture, PCR, and qPCR. mNGS also identified additional species, including *U*. *urealyticum*, and notably, *C. propinquum*, and *S. epidermidis*, both of which were considered as members of normal human microbiota in the culture report. However, *C. propinquum* is also an emerging cause of respiratory infections (14), and some strains are capable of producing urease (15).

Since the output of the iSeq 100 is lower than that of other Illumina (San Diego, CA, USA) instruments (MiniSeq, MiSeq or NextSeq), successful removal of host DNA is important for the detection of microbial DNA. However, the proportion of human reads in our iSeq NGS dataset remained high, in contrast to a previous report (16) in which the same method for microbial enrichment procedure had been used.

Most currently used culture-independent methods require a relatively precise suspicion of the pathogen causing the infection, while mNGS can “blindly” detect all microorganisms present within a clinical sample and assess their load (17). Provided that a sufficient number of bacterial reads are generated by mNGS (a situation which is more likely to occur before the initiation of the antibiotic treatment), the resistance profile (resistome) may be assessed (16–18) in a much quicker fashion than using culture-based methods (11, 12, 19). In some cases, mNGS may also be used for bacterial typing, bypassing the need for multiple PCR or qPCR tests (20). As a hypothesis-free approach, it is also well-suited for detecting new or unexpected pathogens (21). mNGS may provide an advantage over classical methods because it has the potential to predict infection and to help differentiate colonization from infection (22). The mNGS analysis of sequential BAL fluids of the same patient could allow follow-up of the infectious process and provide proof of a microbiological cure.

## Conclusions

Life-threatening complications of *Mycoplasma*/*Ureaplasma* infections following lung or stem-cells transplantations have been reported, but the underlying mechanisms are not fully understood. Detection of *Mycoplasma*/*Ureaplasma* infections require specific molecular tests and growth media that many clinical microbiology laboratories do not perform routinely. It is likely that many infections caused by *Mycoplasma*/*Ureaplasma* remain undocumented.

mNGS as a diagnostic tool in clinical microbiology can help identify and quantify not only pathogens but also commensals, some of which have a pathogenic potential (pathobionts) in immunocompromised patients.

## Materials/Methods

Standard BAL microbiological procedures: 10 μL of the BAL fluid was inoculated on a Columbia Agar with 5% sheep blood (BD, Franklin Lakes, NJ, USA) and a HAEM2 Agar (bioMérieux) for 48 h under a 5% CO_2_ atmosphere at 35 ± 2°C.

*M. hominis/Ureaplasma* spp. cultures and antibiogram: the BAL fluid of the patient was inoculated in the special liquid medium R2 for *Mycoplasma*/*Ureaplasma* and then plated on A7 agar plates (bioMérieux) for culture which were positive after 24 h. The diagnosis was made by optical microscopic observation. The antibiogram in liquid media was carried out by *Mycoplasma* IST2 gallery (bioMérieux), and the results were read at 48 h after the positive control turned positive.

Standard viral culture was performed on cells MRC5, LLC and VERO and incubated 2–3 weeks at 35 ± 2°C.

Multiplex Respiratory Pathogens Taqman Array Card (Thermo Fisher Scientific): customized multiplex PCR cards designed to detect the following pathogens (23): Adenovirus, Bocavirus, CMV, Coronavirus 229E/NL63/HKU1/OC43, HSV1/2, HHV6, VZV, RSV, Enterovirus, Influenza A, Influenza B, hMPV, Parainfluenza virus 1/2/3/4, Rhinovirus, *Bordetella pertussis, Chlamydia psittaci, Chlamydophila pneumoniae, Coxiella burnetii, Legionella pneumophila, Mycoplasma pneumoniae, Pneumocystis jirovecii, Aspergillus fumigatus, Aspergillus flavus, Aspergillus niger*, and *Aspergillus terreus*.

The specific PCRs for *M. hominis, U. urealyticum*, and *U. parvum* were performed by the National Reference Center for *Chlamydia trachomatis* and urogenital *Mycoplasma* species of the University Hospital of Bordeaux, France.

mNGS pipeline: The BAL fluid (400 μL, conserved at −80°C) was mixed with an equal volume of 10 mg/mL Liberase (TL Research grade, Sigma-Aldrich, St. Louis, MO, USA) in Dulbecco's phosphate-buffered saline with MgCl_2_ and CaCl_2_ (D8662, Sigma-Aldrich) and incubated with agitation (1,000 rpm) at 37°C for 1.5 h. The suspension was passed through a 100-μm nylon strainer (Corning), which was washed with 200 μL of the SU buffer from Ultra-Deep Microbiome Prep kit (Molzym, Bremen, Germany). For microbial DNA enrichment, we used the Ultra-Deep Microbiome Prep kit as per manufacturer's recommendations for liquid samples. Negative extraction was performed using 400 μL of the SU buffer (Molzym) instead of the clinical sample. Metagenomic libraries were constructed from extracted DNA using Nextera DNA Flex Library Prep kit (Illumina) and sequenced (2 × 151) on an Illumina iSeq 100 System. Our bioinformatics pipeline included (1) quality filtering (24); (2) elimination of replicate reads; (3) removal of reads matching human genome sequence; and (4) classification of reads using CLARK (25) against the Latest RefSeq/NCBI reference/representative (26) bacterial, archaeal, and fungal genomes, as well as genomes of DNA viruses infecting humans and DNA bacteriophages (selected from https://viralzone.expasy.org). To calculate genome coverage, metagenomic reads classified by CLARK were mapped to corresponding reference genomic sequence using USEARCH 11.0.667 (27) (-ublast -id 0.8 query_cov 0.5 -top_hit_only -strand both -evalue 0.00001). The reads assigned by CLARK to *Mycoplasma* and *Ureaplasma* were queried against the entire NCBI database using the BLASTn (28) tool on the NCBI website (29).

After filtering out the sequencing read pairs matching the human genome, sequencing data were submitted to European Nucleotide Archive (ENA) under study number PRJEB44898.

qPCR/mNGS based quantification of bacteria: The abundance of a given bacterial species was computed from relevant bacterial to human read counts ratio combined with a TaqMan-based quantification of human DNA (30). Obtained values were corrected for genome sizes of reference bacterial genomes.

## Data Availability Statement

The datasets presented in this study can be found in online repository at: https://www.ebi.ac.uk/ena, under study number PRJEB44898.

## Ethics Statement

The Ethics Committee waived the requirement for informed consent for this case report. Written informed consent was not obtained from the individual for the publication of any potentially identifiable data included in this article.

## Author Contributions

CM: microbiological diagnosis and writing. MR: clinical informations and writing. VL and JS: mNGS, correction of the manuscript, and writing. NG: bioinformatics. NL and CK: clinical care of the patient and follow-up. MHa and OV: microbiological diagnosis and correction of the manuscript. DG: clinical care of the patient. MHi: clinical care of the patient and correction of the manuscript. All authors contributed to the article and approved the submitted version.

## Conflict of Interest

The authors declare that the research was conducted in the absence of any commercial or financial relationships that could be construed as a potential conflict of interest.

## References

[1] Sampath R Patel R Cunningham SA Arif S Daly RC Badley AD. Cardiothoracic transplant recipient mycoplasma hominis: an uncommon infection with probable donor transmission. EBioMed. (2017) 19:84–90. 10.1016/j.ebiom.2017.04.026 28438507 PMC5440619

[2] Spiller OB. Emerging pathogenic respiratory mycoplasma hominis infections in lung transplant patients: time to reassesses it's role as a pathogen? EBioMed. (2017) 19:8–9. 10.1016/j.ebiom.2017.05.002 28506624 PMC5440618

[3] Bharat A Cunningham SA Scott Budinger GR Kreisel D DeWet CJ Gelman AE. Disseminated ureaplasma infection as a cause of fatal hyperammonemia in humans. Sci Transl Med. (2015) 7:284re3. 10.1126/scitranslmed.aaa8419 25904745 PMC4677674

[4] Nowbakht C Edwards AR Rodriguez-Buritica DF Luce AM Doshi PB De Golovine A. Two cases of fatal hyperammonemia syndrome due to mycoplasma hominis and ureaplasma urealyticum in immunocompromised patients outside lung transplant recipients. Open Forum Infect Dis. (2019) 6:ofz033. 10.1093/ofid/ofz033 30863787 PMC6405933

[5] Smith M Crews JD Cheek N Srivastava R Appachi E. Hyperammonemic encephalopathy due to ureaplasma parvum infection in an immunocompromised child. Pediatrics. (2019) 144:e20190601. 10.1542/peds.2019-0601 31324704

[6] Matson KM Sonetti DA. Successful treatment of ureaplasma-induced hyperammonemia syndrome post-lung transplant. Transpl Infect Dis. (2019) 21:e13022. 10.1111/tid.13022 30403322

[7] Fernandez R Ratliff A Crabb D Waites KB Bharat A. Ureaplasma transmitted from donor lungs is pathogenic after lung transplantation. Ann Thorac Surg. (2017) 103:670–1. 10.1016/j.athoracsur.2016.09.026 28109354

[8] Smith DG Russell WC Ingledew WJ Thirkell D. Hydrolysis of urea by ureaplasma urealyticum generates a transmembrane potential with resultant aTP synthesis. J Bacteriol. (1993) 175:3253–8. 10.1128/jb.175.11.3253-3258.1993 8501029 PMC204721

[9] Lanao AE Chakraborty RK Pearson-Shaver AL. Mycoplasma infections. In: StatPearls [Internet]. Treasure Island, FL: StatPearls Publishing (2021). Available online at: https://www.ncbi.nlm.nih.gov/books/NBK536927/30725612

[10] Nosotti M Tarsia P Morlacchi LC. Infections after lung transplantation. J Thorac Dis. (2018) 10:3849–68. 10.21037/jtd.2018.05.204 30069386 PMC6051843

[11] Meygret A Le Roy C Renaudin H Bébéar C Pereyre S. Tetracycline and fluoroquinolone resistance in clinical *Ureaplasma* spp. and *Mycoplasma hominis* isolates in France between 2010 and 2015. J Antimicrob Chemother. (2018) 73:2696–703. 10.1093/jac/dky238 29986031

[12] Foschi C Salvo M Galli S Moroni A Cevenini R Marangoni A. Prevalence and antimicrobial resistance of genital mollicutes in italy over a two-year period. New Microbiol. (2018) 41:153–8. 29498739

[13] Trushin MV Chernov VM Gorshkov OV Baranova NB Chernova OA. Atomic force microscopy analysis of DNA extracted from the vegetative cells and the viable, but nonculturable, cells of two mycoplasmas (*Acholeplasma laidlawii* PG8 and *Mycoplasma hominis* PG37). Sci World J. (2010) 10:894–900. 10.1100/tsw.2010.78 20495768 PMC5763845

[14] Díez-Aguilar M Ruiz-Garbajosa P Fernández-Olmos A Guisado P Del Campo R Quereda C. Non-diphtheriae corynebacterium species: an emerging respiratory pathogen. Eur J Clin Microbiol Infect Dis. (2013) 32:769–72. 10.1007/s10096-012-1805-5 23271676

[15] Bernard K Pacheco AL Cunningham I Gill N Burdz T Wiebe D. Emendation of the description of the species corynebacterium propinquum to include strains which produce urease. Int J Syst Evol Microbiol. (2013) 63:2146–54. 10.1099/ijs.0.046979-0 23104363

[16] Leo S Gaïa N Ruppé E Emonet S Girard M Lazarevic V. Detection of bacterial pathogens from broncho-Alveolar lavage by next-Generation sequencing. Int J Mol Sci. (2017) 18:2011. 10.3390/ijms18092011 28930150 PMC5618659

[17] Rossen JWA Friedrich AW Moran-Gilad J. Practical issues in implementing whole-genome-sequencing in routine diagnostic microbiology. Clin Microbiol Infect. (2018) 24:355–60. 10.1016/j.cmi.2017.11.001 29117578

[18] Ruppé E Cherkaoui A Lazarevic V Emonet S Schrenzel J. Establishing genotype-to-phenotype relationships in bacteria causing hospital-acquired pneumonia: a prelude to the application of clinical metagenomics. Antibiotics. (2017) 6:30. 10.3390/antibiotics6040030 29186015 PMC5745473

[19] Valentine-King MA Brown MB. Antibacterial resistance in ureaplasma species and mycoplasma hominis isolates from urine cultures in college-Aged females. Antimicrob Agents Chemother. (2017) 22 61:e01104–17. 10.1128/AAC.01104-17 28827422 PMC5610494

[20] Choutko V Lazarevic V Gaïa N Girard M Renzi G Leo S. Rare case of community-acquired endocarditis caused by neisseria meningitidis assessed by clinical metagenomics. Front Cardiovasc Med. (2019) 6:112. 10.3389/fcvm.2019.00112 31448292 PMC6691042

[21] Gu W Miller S Chiu CY. Clinical metagenomic next-Generation sequencing for pathogen detection. Annu Rev Pathol. (2019) 14:319–38. 10.1146/annurev-pathmechdis-012418-012751 30355154 PMC6345613

[22] Emonet S Lazarevic V Leemann Refondini C Gaïa N Leo S Girard M. Identification of respiratory microbiota markers in ventilator-associated pneumonia. Intensive Care Med. (2019) 45:1082–92. 10.1007/s00134-019-05660-8 31209523 PMC6667422

[23] Steensels D Reynders M Descheemaeker P Curran MD Jacobs F Denis O. Clinical evaluation of a multi-parameter customized respiratory taqMan(®) array card compared to conventional methods in immunocompromised patients. J Clin Virol. (2015) 72:36–41. 10.1016/j.jcv.2015.08.022 26364158 PMC7106552

[24] Bolger AM Lohse M Usadel B. Trimmomatic: a flexible trimmer for illumina sequence data. Bioinformatics. (2014) 30:2114–20. 10.1093/bioinformatics/btu170 24695404 PMC4103590

[25] Ounit R Wanamaker S Close TJ Lonardi S. CLARK: fast and accurate classification of metagenomic and genomic sequences using discriminative k-mers. BMC Genomics. (2015) 16:236. 10.1186/s12864-015-1419-2 25879410 PMC4428112

[26] Pruitt KD Tatusova T Maglott DR. (2007) NCBI reference sequences (RefSeq): a curated non- redundant sequence database of genomes, transcripts and proteins. Nucleic Acids Res. 35:D61–5. 10.1093/nar/gkl842 17130148 PMC1716718

[27] Edgar RC. Search and clustering orders of magnitude faster than BLAST. Bioinformatics. (2010) 26:2460–1. 10.1093/bioinformatics/btq461 20709691

[28] Altschul SF Gish W Miller W Myers EW Lipman DJ. Basic local alignment search tool. J Mol Biol. (1990) 215:403–10. 10.1016/S0022-2836(05)80360-2 2231712

[29] Johnson M Zaretskaya I Raytselis Y Merezhuk Y McGinnis S Madden TL. NCBI BLAST: a better web interface. Nucleic Acids Res. (2008) 36:W5–9. 10.1093/nar/gkn201 18440982 PMC2447716

[30] Lazarevic V Gaïa N Girard M François P Schrenzel J. Comparison of dNA extraction methods in analysis of salivary bacterial communities. PLoS ONE. (2013) 8:e67699. 10.1371/journal.pone.0067699 23844068 PMC3701005

